# Implementation of a Structured Perioperative Checklist-Based Standardization Process and Early Postoperative Outcomes After Bariatric Surgery: A Single-Center Retrospective Pre–Post Cohort Study

**DOI:** 10.3390/jcm15197680

**Published:** 2026-10-04

**Authors:** Sertaç Ata Güler, Mehmet Fatih Özsaray, Turgay Şimşek, Nuh Zafer Cantürk

**Affiliations:** 1 Department of General Surgery, Kocaeli University, 41380 Kocaeli, Turkey; drsataguler@kocaeli.edu.tr (S.A.G.); turgay.simsek@kocaeli.edu.tr (T.Ş.); canturkz@yahoo.com (N.Z.C.); 2 Sakarya Pamukova State Hospital, 54900 Pamukova, Turkey

**Keywords:** bariatric surgery, checklist, perioperative care, postoperative complications, Clavien–Dindo classification, obesity

## Abstract

**Background/Objectives**: Structured perioperative checklists may standardize bariatric surgical care, but their association with early clinical outcomes remains uncertain. This study compared 30-day postoperative outcomes before and after implementation of a structured perioperative checklist and characterized complications by type and Clavien–Dindo severity. **Methods**: This single-center retrospective pre–post cohort study included patients undergoing primary bariatric surgery from June 2024 through December 2025. The checklist was prospectively introduced on 29 July 2024. Cohort identification, data extraction, outcome classification, and comparative analyses were performed retrospectively. Forty patients were included in each cohort. The primary outcome was any 30-day postoperative complication; secondary outcomes included complication severity, intensive care unit admission, and length of hospital stay. Checklist implementation was assessed on the basis of documented completion; item-level adherence to individual perioperative measures and corrective actions prompted by checklist responses were not assessed. Accordingly, the intervention evaluated in this study represents implementation of a structured checklist-based documentation and care-standardization process rather than verified adherence to individual checklist components. **Results**: Complications occurred in 4 of 40 patients (10.0%) in the checklist group and 9 of 40 (22.5%) in the non-checklist group (relative risk, 0.44; 95% confidence interval, 0.15–1.33; *p* = 0.13). Minor complications occurred in 7.5% and 15.0%, respectively, and major complications in 2.5% and 7.5%, respectively. The study was underpowered to reliably detect a between-group difference in the observed magnitude in 30-day complication rates; therefore, this comparison should be interpreted as exploratory. Median hospital stay was shorter in the checklist group (3 days; interquartile range, 2.5–4.0) than in the non-checklist group (4 days; interquartile range, 3.0–6.0; Mann–Whitney U = 398; *p* < 0.001). Intensive care unit admission occurred in 7.5% and 17.5%, respectively (*p* = 0.31). Given the sequential, non-overlapping study periods, the observed difference in length of stay cannot be separated from secular changes in clinical practice, discharge processes, institutional factors, or surgical-team experience. **Conclusions**: Checklist implementation was temporally associated with a shorter postoperative length of stay, whereas the observed reduction in 30-day complications did not reach statistical significance. Because the cohorts were treated during sequential, non-overlapping periods, these differences cannot be attributed independently to checklist implementation. The findings should therefore be considered exploratory and hypothesis-generating.

## 1. Introduction

Obesity is a major global public health concern, with a continuously increasing prevalence worldwide [1]. It is strongly associated with comorbid conditions such as type 2 diabetes mellitus, cardiovascular diseases, hypertension, and certain malignancies, all of which contribute to increased morbidity and mortality. Bariatric surgery has been established as the most effective long-term treatment for severe obesity, providing sustained weight loss and significant improvement in metabolic parameters [2,3]. Despite these benefits, bariatric surgery carries a risk of postoperative complications. Reported complications include bleeding, wound infection, staple-line or anastomotic leakage, respiratory complications, and thromboembolic events, which may occur during the early postoperative period and require timely detection and management [4,5]. Although major complications are relatively infrequent in experienced centers, even minor adverse events may delay mobilization, prolong hospital stay, increase healthcare costs, and reduce patient satisfaction [6]. Postoperative outcomes after bariatric surgery are influenced by patient- and procedure-related risk factors, perioperative care, and provider- and institution-level characteristics, including surgeon and hospital experience [6,7]. Standardizing modifiable components of perioperative care may therefore contribute to improved outcomes.

Enhanced Recovery After Surgery (ERAS) protocols have been widely implemented and are associated with shorter hospitalization and improved postoperative recovery after bariatric surgery [8,9]. The original multicenter study of the World Health Organization (WHO) Surgical Safety Checklist demonstrated reductions in surgical morbidity and mortality after implementation [10]. More recent evidence syntheses indicate that surgical checklists can improve safety processes and may reduce postoperative morbidity, while emphasizing that their effectiveness depends on implementation quality, local adaptation, team engagement, and adherence [11,12].

Building from this evidence, procedure-specific perioperative pathways and checklist-based approaches have been developed for bariatric surgery to address its unique perioperative challenges [13,14]. These checklists aim to support consistent implementation and documentation of evidence-based perioperative practices, such as thromboembolism prophylaxis, infection prevention, and appropriate patient preparation, while also facilitating structured monitoring for postoperative complications.

Although ERAS protocols and bariatric-specific perioperative pathways have demonstrated benefits in postoperative recovery, evidence specifically evaluating the association between structured perioperative checklist implementation and early clinical outcomes in bariatric surgery remains limited [9,14]. In bariatric outcome research, postoperative morbidity should not only be reported as a binary event but should also be characterized according to complication type and severity [15,16,17]. Structured checklists may support teamwork and communication [18], while reductions in postoperative morbidity may improve patient-centered outcomes and reduce healthcare costs [19,20]. Contemporary bariatric perioperative guidelines also emphasize coordinated multidisciplinary care [21,22]. Therefore, this study aimed to evaluate early postoperative outcomes before and after implementation of a structured perioperative checklist in patients undergoing bariatric surgery, with additional assessment of complication type and Clavien–Dindo severity.

## 2. Materials and Methods

### 2.1. Study Design and Setting

This single-center retrospective pre–post cohort study was conducted at the Department of General Surgery, Kocaeli University, Türkiye. The structured perioperative checklist was prospectively introduced into routine clinical practice on 29 July 2024, following approval by the Institutional Ethics Committee of Kocaeli University Faculty of Medicine on 18 July 2024 (approval number: 2024-309). The pre-checklist cohort comprised patients who underwent surgery from 1 June 2024 through 28 July 2024, whereas the post-checklist cohort comprised patients who underwent surgery from 29 July 2024 through 31 December 2025. For patients in the pre-implementation cohort whose surgery preceded ethics committee approval, study-specific written informed consent for retrospective use of clinical data was obtained after approval by contacting the patients and obtaining signed consent in person at the hospital.

All procedures in both periods were performed by the same bariatric surgical team under the supervision of the same senior bariatric surgeon. Surgical care and routine 30-day postoperative follow-up were provided as part of standard clinical practice. Checklist implementation and completion were prospective in the post-implementation period. However, cohort identification, eligibility assessment, extraction of clinical and outcome data, complication classification, and comparative statistical analyses were conducted retrospectively. No checklist-related or other study-specific intervention was performed before ethics committee approval.

### 2.2. Patient Selection and Cohort Allocation

Consecutive patients who underwent primary bariatric or metabolic surgery between June 2024 and December 2025 were screened for eligibility. Patients were allocated to the study groups according to the date of surgery. Patients treated from 1 June 2024 through 28 July 2024 constituted the pre-checklist cohort, whereas those treated from 29 July 2024 through 31 December 2025 constituted the post-checklist cohort.

All consecutive patients undergoing bariatric surgery during the predefined study periods were screened for eligibility; no matching, convenience sampling, fixed-size sampling, or other case-selection procedure was applied. A total of 48 bariatric procedures were performed during the pre-implementation period (1 June–28 July 2024), comprising 26 procedures in June and 22 procedures between 1 and 28 July. The checklist was implemented on 29 July 2024; no bariatric procedures were performed between 29 and 31 July. During the post-implementation period (29 July 2024–31 December 2025), 48 bariatric procedures were performed, with the first post-implementation procedures occurring in August 2024. Thus, the identical numbers screened in the two cohorts arose from the observed source data and were not determined by sampling, matching, or selection of a predetermined number of cases.

**Figure 1:**
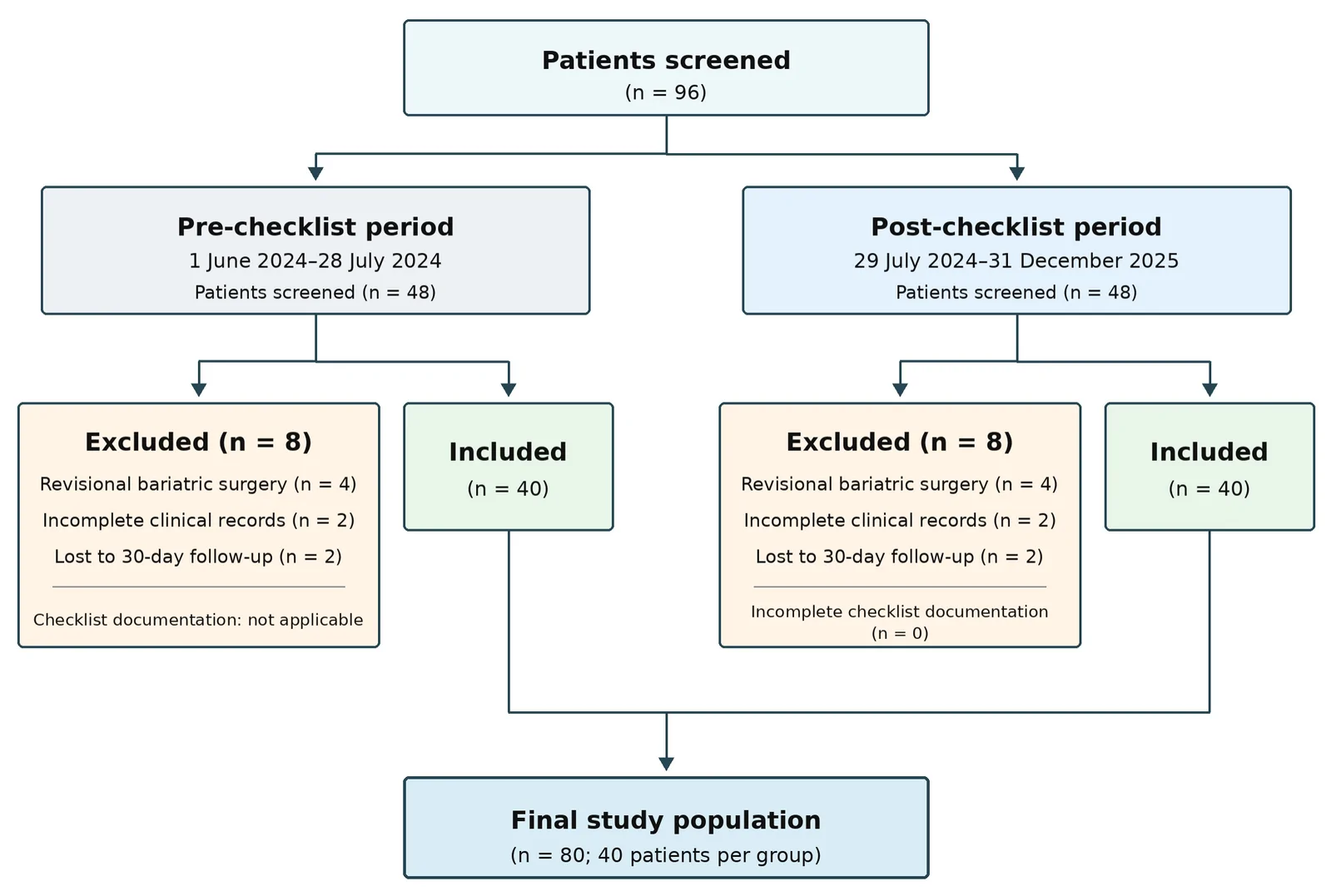
Flow diagram of patient selection and cohort allocation.

A total of 96 patients were screened, including 48 patients in each calendar period. In each period, four patients were excluded because they underwent revisional bariatric surgery, two because of incomplete clinical records, and two because of loss to 30-day postoperative follow-up. Consequently, 40 patients from each period were included in the final analysis. No patient in the post-checklist period was excluded because of missing or incomplete checklist documentation. Cohort eligibility was therefore independent of checklist completion status. The patient selection and cohort allocation process is presented in Figure 1.

### 2.3. Checklist Intervention and Content

The structured perioperative checklist covered the preoperative, intraoperative, postoperative, and discharge phases of care. Preoperative items included assessment of active infection symptoms and drug allergies, review of laboratory and thyroid function tests, discontinuation of anticoagulant medications when applicable, smoking cessation in active smokers, pregnancy testing in female patients, anti-embolism stocking preparation, breathing-exercise training, enoxaparin administration, completion of gastroscopy and other indicated preoperative investigations, and verification of informed consent. Perioperative items included preparation of the operating table and laparoscopic equipment, antibiotic prophylaxis after allergy assessment, pneumatic compression device preparation, patient fixation, bougie placement, preparation for leak testing, and verification of relevant operative documentation. Postoperative items included anti-embolism stockings, enoxaparin administration, mobilization, breathing exercises, drain assessment, urinary catheter removal, initiation and progression of oral intake, laboratory monitoring, dietitian assessment, and contrast fluoroscopy when clinically indicated. Discharge items included assessment for drain and central venous catheter removal, prescription of weight-adjusted enoxaparin for 10 days and paracetamol, recommendation of multivitamin supplementation, and provision of postoperative self-care instructions. The complete itemized checklist and its Yes/No response format are provided in Supplementary Table S1.

The checklist was prospectively completed by the study authors, who were members of the same bariatric surgical team that performed the procedures included in the study. The relevant sections were documented during the corresponding phases of perioperative care. Each applicable item was recorded as either “Yes” or “No.” No separate formal checklist-training program was conducted because the checklist was developed, implemented, and completed directly by the study authors.

For the purposes of the present study, checklist completion was defined as the presence of a documented response for every applicable item. Therefore, the reported completion rate reflects documentation completeness and does not indicate that all recommended measures were performed in every patient. No checklist form contained a missing response, and no patient was excluded because of incomplete checklist documentation.

Item-level response frequencies and the number and type of corrective actions following “No” responses were not prospectively entered into the analytical database and could not be reliably reconstructed for the present analysis. Accordingly, the study evaluates implementation of the checklist as a bundled documentation and care-standardization process rather than adherence to each individual checklist component.

### 2.4. Variables, Outcomes, and Complication Classification

Demographic and perioperative variables included age, sex, body mass index (BMI), smoking status, diabetes mellitus, hypertension, obstructive sleep apnea, American Society of Anesthesiologists (ASA) physical status, previous abdominal surgery, type of bariatric procedure, operative time, postoperative complications, length of hospital stay, and intensive care unit (ICU) admission. Diabetes mellitus, hypertension, obstructive sleep apnea, and previous abdominal surgery were identified from the documented preoperative medical history and electronic medical records. ASA physical status was obtained from the preoperative anesthesia assessment, and operative time was obtained from the operative and anesthesia records. Smoking status was categorized as active smoking versus non-smoking at the time of surgery.

Postoperative complications were defined as any adverse event occurring within 30 days after surgery. Complications were individually extracted from electronic medical records, postoperative progress notes, discharge summaries, emergency department visits, readmission records, radiological reports, endoscopic reports, and operative or interventional records. Each complication was categorized by type, including bleeding, staple-line or anastomotic leakage, wound infection, respiratory complication, thromboembolic event, nausea-vomiting-dehydration requiring medical treatment, and other clinically relevant adverse events.

All complications were graded according to the Clavien–Dindo classification system [17]. Grade I–II complications were classified as minor complications, whereas Grade III or higher complications were classified as major complications. When more than one complication occurred in the same patient, the highest Clavien–Dindo grade was used for patient-level severity analysis. Anonymized patient-level coding underlying the postoperative outcome analyses, including smoking status, postoperative complication status, complication type, Clavien–Dindo grade, minor/major complication classification, ICU admission, and length of hospital stay, is provided in Supplementary Table S2.

The primary outcome was the 30-day postoperative complication rate. Secondary outcomes included complication type, Clavien–Dindo severity, minor and major complication rates, length of hospital stay, and ICU admission.

ICU admission was not considered a postoperative complication per se, because some patients were admitted for short-term postoperative monitoring based on anesthetic evaluation, severe obesity, obstructive sleep apnea risk, or perioperative comorbidity burden despite the absence of a documented surgical complication. However, ICU admission was recorded as a secondary outcome and was also considered when assigning Clavien–Dindo severity in patients with complications requiring ICU-level care.

### 2.5. Statistical Analysis

Statistical analyses were performed using IBM SPSS Statistics for Windows, version 26.0 (IBM Corp., Armonk, NY, USA). Normality of continuous variables was assessed using the Shapiro–Wilk test and inspection of their distributions. Normally distributed continuous variables were summarized as mean ± standard deviation and compared using the independent-samples Student’s *t*-test. Non-normally distributed variables were summarized as median and interquartile range (IQR) and compared using the two-sided Mann–Whitney U test.

Categorical variables were presented as numbers and percentages. Pearson’s chi-square test was used when expected cell counts were adequate, whereas Fisher’s exact test was used for outcomes with small or sparse cell counts. For the primary complication outcome, the relative risk with a log-transformed 95% confidence interval (CI) and the absolute risk difference with a Newcombe 95% CI were calculated. For length of hospital stay, the Hodges–Lehmann location-shift estimate and its 95% confidence interval were reported.

Because this was a retrospective study based on a fixed cohort, the sample size was determined by the number of eligible patients treated during the study period, and no a priori sample-size calculation was performed before data collection. To contextualize the adequacy of the available sample, an exploratory two-sided sample-size estimate was subsequently performed for the primary outcome. Based on the observed 30-day complication rates of 10.0% and 22.5%, corresponding to a Cohen’s h of 0.345, a two-sided alpha level of 0.05, 80% power, and equal group allocation, approximately 132 patients per group (264 patients in total) would have been required to detect a difference in this magnitude. This calculation was used only to inform interpretation and future study planning.

Given the descriptive and exploratory nature of the study, analyses were primarily based on descriptive statistics and univariate comparisons. Because of the limited number of postoperative complication events, multivariable regression and formal effect-modification analyses were not considered statistically reliable. A *p*-value of <0.05 was considered statistically significant.

## 3. Results

During the study period, 96 patients were screened for eligibility, including 48 patients in the pre-checklist period and 48 patients in the post-checklist period. In each cohort, four patients were excluded because of revisional bariatric surgery, two because of incomplete clinical records, and two because of loss to 30-day postoperative follow-up. Thus, 40 patients from each period were included, resulting in a final study population of 80 patients. No post-implementation patient was excluded because of missing or incomplete checklist documentation. The 48 pre-implementation procedures accrued between 1 June and 28 July 2024, whereas the 48 post-implementation procedures accrued between August 2024 and December 2025. Monthly surgical volumes are presented in Table 1. No bariatric procedures were performed between checklist implementation on 29 July 2024 and the end of that month.

All operations in both periods were performed by the same bariatric surgical team under the supervision of the same senior bariatric surgeon. No formal changes in the written surgical or perioperative care protocols were documented during the study period other than implementation of the structured checklist. Nevertheless, because the cohorts were treated during sequential calendar periods, unmeasured temporal changes in clinical practice cannot be excluded.

### 3.1. Baseline Characteristics

Baseline demographic and perioperative characteristics are presented in Table 2. No statistically significant between-group differences were observed in age, sex, BMI, active smoking, diabetes mellitus, hypertension, obstructive sleep apnea, ASA physical status, previous abdominal surgery, procedure type, or operative time.

Diabetes mellitus was present in 11 patients (27.5%) in the checklist group and 13 patients (32.5%) in the non-checklist group (*p* = 0.63). Hypertension was present in 16 (40.0%) and 18 (45.0%) patients, respectively (*p* = 0.65), and obstructive sleep apnea was present in 10 (25.0%) and 12 (30.0%) patients, respectively (*p* = 0.62). Operative time was 78.6 ± 18.4 min in the checklist group and 81.2 ± 20.1 min in the non-checklist group (*p* = 0.55).

Checklist documentation was complete for all 40 patients in the post-implementation cohort, with every applicable item recorded as either “Yes” or “No.” Accordingly, the documentation-completion rate was 100%. This finding represents complete checklist documentation and should not be interpreted as 100% adherence to every recommended perioperative measure. Item-level response frequencies and corrective actions were not available for analysis.

### 3.2. Postoperative Outcomes and Clavien–Dindo Severity

Postoperative outcomes and complication severity are summarized in Table 3. Overall 30-day postoperative complications occurred in 4 patients (10.0%) in the checklist group and 9 patients (22.5%) in the non-checklist group. This corresponded to a relative risk of 0.44 (95% CI, 0.15–1.33) and an absolute risk difference of −12.5 percentage points (95% CI, −28.7 to 4.1; *p* = 0.13). Because both confidence intervals included the null value, the numerical difference remains inconclusive.

Minor complications occurred in 3 patients (7.5%) in the checklist group and 6 patients (15.0%) in the non-checklist group, whereas major complications occurred in 1 patient (2.5%) and 3 patients (7.5%), respectively. Neither difference was statistically significant.

The detailed distribution of complication types and Clavien–Dindo grades is presented in Table 4. The most frequent complications were bleeding, wound infection, and respiratory complications. Staple-line or anastomotic leakage was observed in one patient in the non-checklist group. The checklist group had no leakage, thromboembolic event, or Grade IV complication. The only major complication in the checklist group was bleeding requiring surgical intervention, whereas major complications in the non-checklist group included leakage requiring reoperation or drainage, bleeding requiring endoscopic or radiological intervention, and respiratory deterioration requiring ICU-level respiratory support. The complete anonymized patient-level coding underlying these analyses is provided in Supplementary Table S2.

ICU admission occurred in 3 patients (7.5%) in the checklist group and 7 patients (17.5%) in the non-checklist group (Fisher’s exact *p* = 0.31). Some ICU admissions were undertaken for precautionary postoperative monitoring rather than for an established surgical complication.

Length of hospital stay was shorter in the checklist group, with a median of 3 days (IQR, 2.5–4.0), compared with 4 days (IQR, 3.0–6.0) in the non-checklist group (Mann–Whitney U = 398; *p* < 0.001). The Hodges–Lehmann estimate indicated a 1-day shorter hospital stay in the checklist group (location shift, −1 day; 95% CI, −2 to −1 days).

### 3.3. Descriptive Comparison According to 30-Day Postoperative Complication Status

An exploratory descriptive comparison of patients with and without 30-day postoperative complications is presented in Table 5. ICU admission was excluded from this comparison because it may occur as a consequence of a postoperative complication and should not be interpreted as a baseline or preoperative risk factor. Because only 13 patients experienced a complication, these comparisons were not used to infer independent associations, predictors, or effect modification.

## 4. Discussion

### 4.1. Principal Findings

The principal finding of this study was a temporal association between structured perioperative checklist implementation and shorter hospital stay; however, the sequential pre–post design does not allow this difference to be attributed independently to checklist implementation. Median length of stay was 3 days in the checklist group and 4 days in the non-checklist group. However, this association should not be interpreted as direct evidence of faster clinical recovery or as an effect attributable solely to checklist implementation, because postoperative management, discharge planning, organizational factors, and secular changes in discharge practice may also have contributed.

The overall 30-day complication rate was numerically lower in the checklist group than in the non-checklist group (10.0% vs. 22.5%). The relative risk was 0.44 (95% CI, 0.15–1.33), and the absolute risk difference was −12.5 percentage points (95% CI, −28.7 to 4.1). The confidence intervals included the null value, and the study was underpowered for the primary complication outcome. Therefore, the observed difference remains inconclusive and should not be interpreted as evidence that checklist implementation prevented postoperative morbidity. Complications were individually categorized and graded according to the Clavien–Dindo classification, but the small number of events precluded reliable severity-specific, subgroup, and adjusted analyses.

### 4.2. Interpretation in the Context of Existing Literature

The concept of structured perioperative care has gained increasing importance in recent years. ERAS protocols have been shown to improve postoperative outcomes by reducing surgical stress, optimizing recovery, and minimizing complications [8,9]. Similarly, surgical safety checklists have been widely adopted as effective tools to standardize perioperative processes and improve patient safety. The WHO Surgical Safety Checklist has been demonstrated to significantly reduce morbidity and mortality across a wide range of surgical procedures [10]. The importance of standardized complication reporting has also been emphasized in recent bariatric cohorts. Stryhn et al. evaluated 1600 patients undergoing sleeve gastrectomy within an enhanced recovery after bariatric surgery framework and reported 30-day and 90-day complications using the Clavien–Dindo classification [23]. Similarly, Husain et al. analyzed 30-day postoperative complications after bariatric surgery according to Clavien–Dindo severity and highlighted the relevance of patient- and procedure-related risk factors in postoperative risk assessment [24]. These findings support detailed reporting of postoperative complications according to both type and severity in bariatric outcome studies. Our findings should also be interpreted in the context of previous prospective investigations evaluating structured perioperative tools in bariatric surgery. Seip et al. developed a standardized Readiness for Discharge (RFD) checklist using prospectively collected data derived from a randomized clinical trial and demonstrated that structured checklist-based assessment improved the objective evaluation of postoperative recovery and discharge readiness [13]. Although the RFD checklist focused primarily on discharge assessment rather than prevention of postoperative complications, both studies support the concept that standardized perioperative assessment tools may improve consistency of postoperative management. Unlike the RFD study, which evaluated discharge readiness, our study evaluated a comprehensive perioperative checklist covering the preoperative, intraoperative, postoperative, and discharge phases, with 30-day postoperative complications as the primary outcome and hospital stay as a secondary outcome. Therefore, our findings complement rather than duplicate the existing literature and further support the potential role of structured perioperative checklists in bariatric surgery.

In bariatric surgery, procedure-specific checklists aim to address the unique perioperative risks associated with obese patients. These include increased risk of thromboembolism, respiratory complications, and technical challenges during surgery [14,21,22]. The checklist covered the preoperative, intraoperative, postoperative, and discharge phases and provided a structured format for documenting perioperative measures. All forms were complete at the documentation level. However, complete documentation does not establish complete adherence to every recommended intervention. Because item-level responses and corrective actions were not available for analysis, the present study cannot determine whether the observed findings were related to checklist use itself, improved adherence to individual measures, greater standardization of care, increased team attention, or more structured documentation.

Although the difference in postoperative length of stay was statistically significant, this finding warrants particularly cautious interpretation. Length of stay is influenced not only by postoperative clinical recovery but also by discharge practices, bed availability, institutional workflow, and other time-dependent factors that were not captured in our dataset. Because the pre- and post-implementation cohorts occupied sequential, non-overlapping calendar periods, the association between checklist implementation and length of stay cannot be disentangled from secular changes occurring over time. Accordingly, the shorter length of stay observed in the post-implementation cohort should be interpreted as a temporal association rather than evidence of an independent effect of checklist implementation. ICU admission remained a descriptive secondary outcome because some admissions were undertaken for precautionary monitoring.

Checklists may support standardization, communication, and coordination within multidisciplinary perioperative teams [11,12,18]. Nevertheless, these mechanisms were not directly measured in the present study. Because item-level adherence and corrective actions were not available, the study cannot distinguish the contribution of checklist use from increased team attention, structured documentation, accumulated clinical experience, or other temporal changes occurring between the two periods.

### 4.3. Clinical Implications

From a clinical perspective, accurate characterization of postoperative complications is essential. Even minor complications can negatively affect patient recovery, prolong hospitalization, and increase healthcare costs [19,20]. Structured perioperative checklists may provide a practical framework for standardizing perioperative documentation, monitoring, and communication regarding postoperative morbidity. However, the present study does not establish whether checklist implementation improved adherence to individual preventive or therapeutic measures.

### 4.4. Strengths and Limitations

The present study has several strengths. All procedures were performed by the same bariatric surgical team under the supervision of the same senior bariatric surgeon, thereby limiting, although not eliminating, provider-related variability. Postoperative adverse events were individually extracted, categorized by type, and graded according to the Clavien–Dindo classification. Additional baseline and perioperative variables, including diabetes mellitus, hypertension, obstructive sleep apnea, ASA physical status, previous abdominal surgery, and operative time, were included in the baseline analysis. Furthermore, the numbers and reasons for exclusion were identical in the pre-checklist and post-checklist periods, and no patient was excluded because of incomplete checklist documentation.

Several limitations should be acknowledged. First, the retrospective sequential pre–post design does not permit causal inference and is inherently vulnerable to temporal confounding and secular trends. An important additional source of temporal confounding is the potential effect of increasing surgical-team experience over time. Although the same surgical team performed procedures during both study periods, the sequential design inherently placed patients in the post-implementation cohort later in the team’s cumulative experience. Improvements in operative workflow, perioperative decision-making, postoperative management, and discharge planning may therefore have occurred independently of checklist implementation. This issue is particularly relevant given the short pre-implementation period and substantially longer post-implementation period. The present design cannot disentangle experience-related improvements or other secular changes from associations with checklist implementation.

The markedly different cohort-accrual periods introduce additional temporal and organizational heterogeneity. Although all consecutive bariatric procedures were screened and no fixed-size or convenience sampling was applied, 48 procedures accrued during the approximately two-month pre-implementation period, whereas 48 procedures accrued over approximately 17 months after implementation. Surgical volume therefore decreased substantially during the post-implementation period. The retrospective dataset was not designed to determine the reasons for this change, and unmeasured changes in institutional activity, referral patterns, scheduling, or other organizational factors may have accompanied it. This pronounced temporal and volume asymmetry further increases the potential for secular and organizational confounding and limits attribution of observed outcome differences specifically to checklist implementation.

Second, the sample size was small and only 13 patients experienced a postoperative complication. The exploratory sample-size estimate indicated that approximately 264 patients would have been required to detect the observed absolute difference in complication rates with 80% power. The present study was therefore underpowered for the primary complication outcome, and the absence of statistical significance should not be interpreted as evidence of equivalence between the groups.

Third, although several additional baseline and perioperative characteristics were included, the limited number of events precluded reliable multivariable adjustment without substantial overfitting. Residual confounding by other measured or unmeasured patient-, procedure-, provider-, and institution-related factors therefore cannot be excluded.

Fourth, checklist completion was assessed at the documentation level. Item-level adherence and the frequency and nature of corrective actions following negative responses were not available for analysis. Because the checklist was completed by members of the study team and implementation was not independently audited, documentation and performance bias are also possible. The study cannot distinguish the effects of checklist use, adherence to individual measures, structured documentation, and increased team attention.

Fifth, length of hospital stay is susceptible to nonclinical and organizational influences. Bed availability, institutional pressure to discharge patients, clinician judgment, patient preferences, social circumstances, and temporal changes in discharge practice were not recorded. These factors may have contributed to the observed difference in hospital stay.

Finally, 16 of the 96 screened patients were excluded. Although the numbers and reasons for exclusion were identical in the two periods and no patient was excluded because of checklist documentation, exclusion of patients with incomplete records or follow-up may still have introduced selection bias. The study was also conducted at a single institution using a locally developed checklist that has not been externally validated, limiting the generalizability of the findings.

### 4.5. Future Directions

Future studies should use prospective, adequately powered, multicenter designs and should assess not only checklist completion but also item-level adherence, implementation fidelity, and corrective actions prompted by checklist responses. Such studies should incorporate relevant patient- and procedure-level confounders and evaluate whether shorter hospitalization is accompanied by improvements in patient-centered recovery, readmission, and post-discharge complication rates.

## 5. Conclusions

In this single-center retrospective pre–post cohort, implementation of a structured perioperative checklist-based standardization process was temporally associated with a shorter postoperative length of stay, while the observed reduction in 30-day complications did not reach statistical significance. The sequential study periods, substantial differences in cohort-accrual duration and surgical volume, potential secular changes, and increasing surgical-team experience prevent attribution of these findings independently to checklist implementation. Moreover, checklist completion reflected documentation of a structured perioperative process rather than verified adherence to individual clinical measures. The findings should therefore be interpreted as exploratory and hypothesis-generating. Prospective, adequately powered, multicenter studies with contemporaneous controls and assessment of item-level checklist adherence are needed to determine whether structured perioperative checklist implementation independently improves outcomes after bariatric surgery.

## Figures and Tables

**Table 1:** Monthly Bariatric Surgical Volume During the Study Period.

Month	*n*
June 2024	26
July 2024 *	22
August 2024	10
September 2024	6
October 2024	4
November 2024	3
December 2024	3
January 2025	3
February 2025	2
March 2025	3
April 2025	2
May 2025	2
June 2025	2
July 2025	1
August 2025	2
September 2025	1
October 2025	2
November 2025	1
December 2025	1

* All 22 bariatric procedures performed in July 2024 occurred between 1 and 28 July and therefore belonged to the pre-implementation cohort. The checklist was implemented on 29 July 2024, and no bariatric procedures were performed between 29 and 31 July; consequently, the first post-implementation procedures occurred in August 2024.

**Table 2:** Baseline demographic and perioperative characteristics of the study population.

Variable	Checklist Group (*n* = 40)	Non-Checklist Group (*n* = 40)	*p*-Value
Age, years	38.6 ± 9.4	39.8 ± 10.1	0.58
Female sex, *n* (%)	26 (65.0)	25 (62.5)	0.81
BMI, kg/m^2^	42.3 ± 5.1	43.1 ± 5.6	0.47
Active smoking, *n* (%)	9 (22.5)	12 (30.0)	0.45
Diabetes mellitus, *n* (%)	11 (27.5)	13 (32.5)	0.63
Hypertension, *n* (%)	16 (40.0)	18 (45.0)	0.65
Obstructive sleep apnea, *n* (%)	10 (25.0)	12 (30.0)	0.62
ASA physical status, *n* (%)			0.64
ASA II	27 (67.5)	25 (62.5)	
ASA III	13 (32.5)	15 (37.5)	
Previous abdominal surgery, *n* (%)	9 (22.5)	11 (27.5)	0.61
Type of bariatric procedure, *n* (%)			0.78
Sleeve gastrectomy	32 (80.0)	31 (77.5)	
Gastric bypass	8 (20.0)	9 (22.5)	
Operative time, min	78.6 ± 18.4	81.2 ± 20.1	0.55

Data are presented as mean ± standard deviation or *n* (%), as appropriate. Continuous variables were compared using the independent-samples Student’s *t*-test, and categorical variables were compared using Pearson’s chi-square test. The *p*-values for ASA physical status and procedure type represent overall between-group comparisons.

**Table 3:** Comparison of 30-day postoperative outcomes and complication severity between checklist and non-checklist groups.

Outcome	Checklist Group (*n* = 40)	Non-Checklist Group (*n* = 40)	*p*-Value
Any 30-day complication, *n* (%)	4 (10.0)	9 (22.5)	0.13
Minor complication, Clavien–Dindo Grade I–II, *n* (%)	3 (7.5)	6 (15.0)	0.48
Major complication, Clavien–Dindo Grade III or higher, *n* (%)	1 (2.5)	3 (7.5)	0.62
ICU admission, *n* (%)	3 (7.5)	7 (17.5)	0.31
Length of hospital stay, days, median (IQR)	3.0 (2.5–4.0)	4.0 (3.0–6.0)	<0.001

Data are presented as *n* (%) unless otherwise specified. The overall 30-day complication rate was compared using Pearson’s chi-square test. Minor and major complications and ICU admission were compared using Fisher’s exact test because of sparse cell counts. Length of hospital stay is presented as median (interquartile range) and was compared using the two-sided Mann–Whitney U test. Individual Clavien–Dindo grades are reported descriptively in Table 4 and were not subjected to grade-by-grade inferential comparisons because of the small number of events.

**Table 4:** Patient-level details of 30-day postoperative complications.

Group	Complication Type	Clavien–Dindo Grade	Minor/Major	Management
Checklist	Wound infection	I	Minor	Local wound care
Checklist	Nausea-vomiting-dehydration	II	Minor	IV fluid and antiemetic treatment
Checklist	Respiratory complication	II	Minor	Oxygen support and medical treatment
Checklist	Bleeding	IIIb	Major	Surgical intervention
Non-checklist	Bleeding	II	Minor	Blood transfusion
Non-checklist	Staple-line/anastomotic leakage	IIIb	Major	Reoperation/drainage
Non-checklist	Wound infection	I	Minor	Local wound care
Non-checklist	Respiratory complication	II	Minor	Oxygen support and antibiotics
Non-checklist	Thromboembolic event	II	Minor	Therapeutic anticoagulation
Non-checklist	Nausea-vomiting-dehydration	II	Minor	IV fluid and antiemetic treatment
Non-checklist	Bleeding	IIIa	Major	Endoscopic or radiological intervention
Non-checklist	Wound infection	II	Minor	Antibiotic treatment
Non-checklist	Respiratory complication	IVa	Major	ICU-level respiratory support

**Table 5:** Exploratory descriptive comparison of patients with and without 30-day postoperative complications.

Variable	Complication (+) (*n* = 13)	Complication (−) (*n* = 67)	*p*-Value
Age (years)	41.2 ± 10.5	38.9 ± 9.6	0.38
Female sex, *n* (%)	8 (61.5)	43 (64.2)	1.00
BMI (kg/m^2^)	44.1 ± 5.8	42.6 ± 5.3	0.29
Checklist use, *n* (%)	4 (30.8)	36 (53.7)	0.22

Age and BMI were compared using the independent-samples Student’s *t*-test. Categorical variables were compared using Fisher’s exact test. All comparisons were exploratory and unadjusted.

## Data Availability

The data presented in this study are not publicly available due to privacy and ethical restrictions, as they contain patient-level clinical data. The data are available from the corresponding author upon reasonable request and subject to applicable ethical approval.

## Supplementary Materials

The following supporting information can be downloaded at: https://www.mdpi.com/article/10.3390/jcm15197680/s1, Table S1: Structured Perioperative Bariatric Surgery Checklist; Table S2: Anonymized patient-level coding of smoking status, postoperative complications, Clavien–Dindo grade, ICU admission, and length of hospital stay.

## Abbreviations

ASA	American Society of Anesthesiologists
BMI	Body mass index
CI	Confidence interval
ERAS	Enhanced Recovery After Surgery
ICU	Intensive care unit
IQR	Interquartile range
WHO	World Health Organization
