# Supplementary material for: Implementation of a Structured Perioperative Checklist-Based Standardization Process and Early Postoperative Outcomes After Bariatric Surgery: A Single-Center Retrospective Pre–Post Cohort Study
Source: J Clin Med. 2026 Oct 4;15(19):7680. doi: 10.3390/jcm15197680 (PMC13665956; doi:10.3390/jcm15197680)
Supplement: Supplementary file 1 [file jcm-15-07680-s001.zip › jcm-4511219-supplementary.pdf]

Supplementary Table S1

Structured Perioperative Bariatric Surgery Checklist

PRE-OPERATIVE			PERI-OPERATIVE		
	YES	NO		YES	NO
Oral wound/herpes lesion check			Surgical table check		
Nasal discharge or cough present?			Laparoscopic instruments sterilized/prepared?		
Any drug allergy present?			Methylene blue preparation for leak test (minimum 100 cc in catheter-tip syringe)		
Anticoagulants (Ecopirin, Coraspirin, Plavix, Xarelto, Warfarin, Coumadin) discontinued at least 1 week before surgery?			Laparoscopic system video recording prepared		
If active smoker, smoking cessation at least 2 weeks before surgery?			Laparoscopic insufflation gas level checked (Level 1–2–3–4)		
Beta-hCG test checked in female patients			Thyroid function test (TFT) result review if pending		
Review of laboratory test results			Follow-up of pending consultations if present		
Thyroid function test (TFT) review if pending			Pneumatic compression device prepared		
Anti-embolism stocking check			Anesthesia consent signed and present in file		
Triflow breathing exercise training			Surgical informed consent signed and present in file		
Enoxaparin administration			Anti-embolism stockings applied		
Gastroscopy completed			Prophylactic antibiotic administration after allergy assessment		
If H. pylori positive, treatment completed?			Verification of appropriate patient fixation on operating table		
Colonoscopy assessment in patients >50 years			Verification that no orogastric or nasogastric tube is present		
Total abdominal ultrasonography completed			Bougie placement		
In female patients: breast ultrasonography and/or mammography according to age			Anesthesia form signed and present in file		
Medical board report available			Surgical informed consent signed and present in file		
Preoperative abdominal skin preparation completed					

**POST-OPERATIVE**											
**0 hour**			**8th hour**			**Day 1**			**Day 2**		
	**Yes**	**No**		**Yes**	**No**		**Yes**	**No**		**Yes**	**No**
Anti-embolism stockings			Mobilization			Urinary catheter removed?			Controlled oral intake initiated with small sips (R1 trial)		
			Drain check			Mobilization			Drain check		
			Anti-embolism stockings continued			Drain check			Dietitian consultation completed		
			Enoxaparin administration			Anti-embolism stockings			Hemoglobin and biochemical tests obtained		
			Triflow breathing exercises performed			Enoxaparin administration			Contrast fluoroscopy performed if clinically indicated		
			Dressing control (without opening)			Triflow breathing exercises performed					
						Hemoglobin and biochemical tests obtained					

**DISCHARGE**		
	**Yes**	**No**
Drain removed?		
Central venous pressure (CVP) catheter removed?		
Enoxaparin prescribed for 10 days according to patient weight?		
Paracetamol prescribed?		
Multivitamin supplementation recommended?		
Patient informed that showering is permitted?		

Supplementary Table S2. Anonymized Patient-level coding of smoking status, postoperative complications, Clavien-Dindo grade, ICU admission, and length of hospital stay

Patient ID	Group	Smoking status	Complication	Complication type	Clavien-Dindo grade	Minor complication	Major complication	ICU admission	LOS, days
C01	Checklist	Non-smoker	Yes	Wound infection	I	Yes	No	No	4
C02	Checklist	Current smoker	Yes	Nausea-vomiting-dehydration	II	Yes	No	No	4
C03	Checklist	Non-smoker	Yes	Respiratory complication	II	Yes	No	No	5
C04	Checklist	Non-smoker	Yes	Bleeding	IIIb	No	Yes	Yes	7
C05	Checklist	Current smoker	No	None	None	No	No	Yes	2
C06	Checklist	Non-smoker	No	None	None	No	No	Yes	2
C07	Checklist	Non-smoker	No	None	None	No	No	No	2
C08	Checklist	Current smoker	No	None	None	No	No	No	2
C09	Checklist	Non-smoker	No	None	None	No	No	No	2
C10	Checklist	Non-smoker	No	None	None	No	No	No	2
C11	Checklist	Non-smoker	No	None	None	No	No	No	2
C12	Checklist	Current smoker	No	None	None	No	No	No	2
C13	Checklist	Non-smoker	No	None	None	No	No	No	2
C14	Checklist	Non-smoker	No	None	None	No	No	No	2
C15	Checklist	Non-smoker	No	None	None	No	No	No	3
C16	Checklist	Current smoker	No	None	None	No	No	No	3
C17	Checklist	Non-smoker	No	None	None	No	No	No	3
C18	Checklist	Non-smoker	No	None	None	No	No	No	3
C19	Checklist	Non-smoker	No	None	None	No	No	No	3
C20	Checklist	Current smoker	No	None	None	No	No	No	3
C21	Checklist	Non-smoker	No	None	None	No	No	No	3
C22	Checklist	Non-smoker	No	None	None	No	No	No	3
C23	Checklist	Non-smoker	No	None	None	No	No	No	3
C24	Checklist	Current smoker	No	None	None	No	No	No	3
C25	Checklist	Non-smoker	No	None	None	No	No	No	3
C26	Checklist	Non-smoker	No	None	None	No	No	No	3
C27	Checklist	Non-smoker	No	None	None	No	No	No	3
C28	Checklist	Current smoker	No	None	None	No	No	No	3
C29	Checklist	Non-smoker	No	None	None	No	No	No	3
C30	Checklist	Non-smoker	No	None	None	No	No	No	3
C31	Checklist	Non-smoker	No	None	None	No	No	No	3
C32	Checklist	Current smoker	No	None	None	No	No	No	3
C33	Checklist	Non-smoker	No	None	None	No	No	No	3
C34	Checklist	Non-smoker	No	None	None	No	No	No	4
C35	Checklist	Non-smoker	No	None	None	No	No	No	4
C36	Checklist	Non-smoker	No	None	None	No	No	No	4
C37	Checklist	Non-smoker	No	None	None	No	No	No	4
C38	Checklist	Non-smoker	No	None	None	No	No	No	5
C39	Checklist	Non-smoker	No	None	None	No	No	No	5
C40	Checklist	Non-smoker	No	None	None	No	No	No	5
NC01	Non-checklist	Current smoker	Yes	Bleeding	II	Yes	No	No	5
NC02	Non-checklist	Current smoker	Yes	Staple-line/anastomotic leakage	IIIb	No	Yes	Yes	9
NC03	Non-checklist	Non-smoker	Yes	Wound infection	I	Yes	No	No	5
NC04	Non-checklist	Non-smoker	Yes	Respiratory complication	II	Yes	No	Yes	6
NC05	Non-checklist	Current smoker	Yes	Thromboembolic event	II	Yes	No	Yes	7
NC06	Non-checklist	Non-smoker	Yes	Nausea-vomiting-dehydration	II	Yes	No	No	5
NC07	Non-checklist	Non-smoker	Yes	Bleeding	IIIa	No	Yes	Yes	8
NC08	Non-checklist	Non-smoker	Yes	Wound infection	II	Yes	No	No	6
NC09	Non-checklist	Current smoker	Yes	Respiratory complication	IVa	No	Yes	Yes	10
NC10	Non-checklist	Non-smoker	No	None	None	No	No	Yes	6
NC11	Non-checklist	Current smoker	No	None	None	No	No	Yes	6

Patient ID	Group	Smoking status	Complication	Complication type	Clavien-Dindo grade	Minor complication	Major complication	ICU admission	LOS, days
NC12	Non-checklist	Non-smoker	No	None	None	No	No	No	6
NC13	Non-checklist	Non-smoker	No	None	None	No	No	No	6
NC14	Non-checklist	Current smoker	No	None	None	No	No	No	6
NC15	Non-checklist	Non-smoker	No	None	None	No	No	No	6
NC16	Non-checklist	Non-smoker	No	None	None	No	No	No	6
NC17	Non-checklist	Non-smoker	No	None	None	No	No	No	4
NC18	Non-checklist	Current smoker	No	None	None	No	No	No	4
NC19	Non-checklist	Non-smoker	No	None	None	No	No	No	4
NC20	Non-checklist	Non-smoker	No	None	None	No	No	No	4
NC21	Non-checklist	Non-smoker	No	None	None	No	No	No	4
NC22	Non-checklist	Current smoker	No	None	None	No	No	No	4
NC23	Non-checklist	Non-smoker	No	None	None	No	No	No	4
NC24	Non-checklist	Non-smoker	No	None	None	No	No	No	4
NC25	Non-checklist	Non-smoker	No	None	None	No	No	No	4
NC26	Non-checklist	Current smoker	No	None	None	No	No	No	3
NC27	Non-checklist	Non-smoker	No	None	None	No	No	No	3
NC28	Non-checklist	Non-smoker	No	None	None	No	No	No	3
NC29	Non-checklist	Non-smoker	No	None	None	No	No	No	3
NC30	Non-checklist	Current smoker	No	None	None	No	No	No	3
NC31	Non-checklist	Non-smoker	No	None	None	No	No	No	3
NC32	Non-checklist	Non-smoker	No	None	None	No	No	No	3
NC33	Non-checklist	Non-smoker	No	None	None	No	No	No	3
NC34	Non-checklist	Current smoker	No	None	None	No	No	No	3
NC35	Non-checklist	Non-smoker	No	None	None	No	No	No	3
NC36	Non-checklist	Non-smoker	No	None	None	No	No	No	3
NC37	Non-checklist	Non-smoker	No	None	None	No	No	No	3
NC38	Non-checklist	Current smoker	No	None	None	No	No	No	3
NC39	Non-checklist	Non-smoker	No	None	None	No	No	No	3
NC40	Non-checklist	Non-smoker	No	None	None	No	No	No	3

C = checklist group; NC = non-checklist group; ICU = intensive care unit. For patients without complications, complication type and Clavien-Dindo grade were coded as None. Minor complications were defined as Clavien-Dindo Grade I-II and major complications as Grade III or higher.
